# Supplementary material for: Truncating tau reveals different pathophysiological actions of oligomers in single neurons
Source: Commun Biol. 2021 Nov 4;4:1265. doi: 10.1038/s42003-021-02791-x (PMC8569149; doi:10.1038/s42003-021-02791-x)
Supplement: Supplementary file 2 — Supplementary Information [file 42003_2021_2791_MOESM2_ESM.pdf]

**Supplementary Information**

**Peak Input resistance**

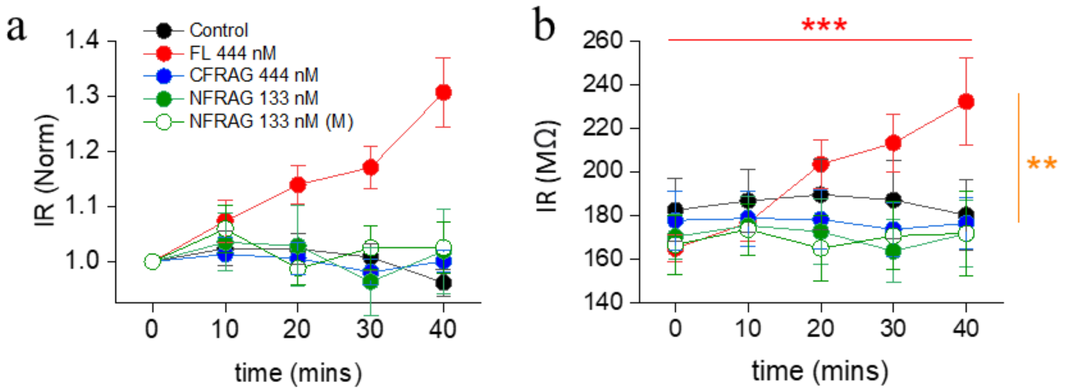


**Supplementary Figure 1: Changes to peak input resistance over time.** Data for all 5 conditions is plotted over time (mean ± SEM) both normalised (**a**), to allow changes over time to be clearly observed and non-normalised (**b**). 2-way ANOVA analysis was carried out on non-normalised data for all groups (V) Vehicle; (FL) Full length oTau; (C) CFRAG oTau; (N) NFRAG oTau; (Nm) NFRAG mTau. There was no statistical difference at time 0 between any of the conditions, in agreement with the manuscript. A significant difference was observed between the different conditions over time ($F$ (4, 275) = 3.513), $p$ =0.0081. Error bars represent standard error of the mean (SEM).

| **30 mins** | V | FL | C | N | Nm |
|---|---|---|---|---|---|
| V | | * | ns | ns | ns |
| FL | | | * | * | * |
| C | | | | ns | ns |
| N | | | | | ns |
| Nm | | | | | |

| **40 mins** | V | FL | C | N | Nm |
|---|---|---|---|---|---|
| V | | ** | ns | ns | ns |
| FL | | | ** | ** | ** |
| C | | | | ns | ns |
| N | | | | | ns |
| Nm | | | | | |

**Supplementary Table 1:** Multiple comparisons between conditions at each timepoint were carried out and the results at 30 and 40 minutes post-whole cell breakthrough.

| | FL oTau |
|---|---|
| 0 mins vs 30 mins | * |
| 0 mins vs 40 mins | *** |

**Supplementary Table 2:** The only condition found to change significantly over time was FL oTau 444 nM. This data is in full agreement with the conclusions in the manuscript.

**Action potential height**

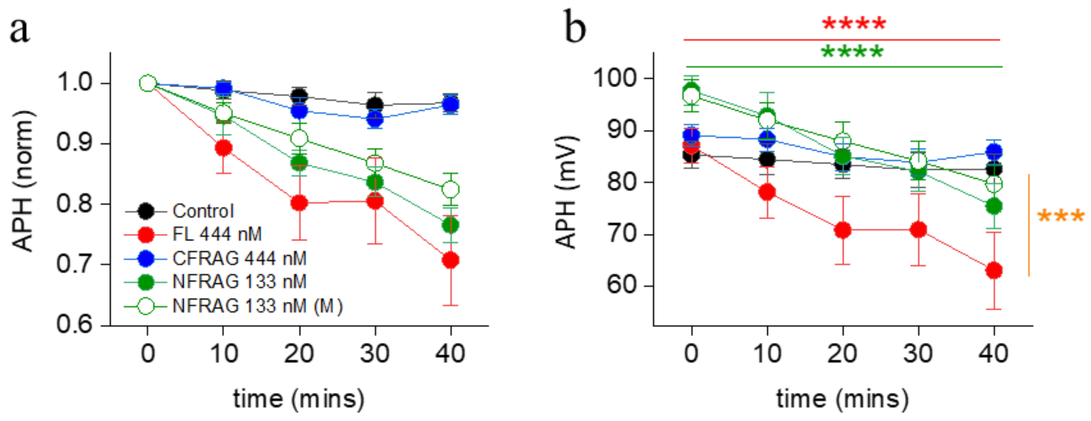


**Supplementary Figure 2: Changes to action potential height over time.** Data for all 5 conditions is plotted over time (mean ± SEM) both normalised (**a**), to allow changes over time to be clearly observed and non-normalised (**b**). 2-way ANOVA analysis was carried out on non-normalised data for all groups (V) Vehicle; (FL) Full length oTau; (C) CFRAG oTau; (N) NFRAG oTau; (Nm) NFRAG mTau. There was no statistical difference at time 0 between any of the conditions, in agreement with the manuscript. A significant difference was observed between the different conditions ($F (4, 275) = 10.26$, $p < 0.0001$) and over time ($F (4, 275) = 9.456$, $p < 0.0001$). Error bars represent standard error of the mean (SEM).

| **30 mins** | V | FL | C | N | Nm |
|---|---|---|---|---|---|
| V | | * | ns | ns | ns |
| FL | | | * | * | * |
| C | | | | ns | ns |
| N | | | | | ns |
| Nm | | | | | |

| **40 mins** | V | FL | C | N | Nm |
|---|---|---|---|---|---|
| V | | *** | ns | ns | ns |
| FL | | | **** | * | ** |
| C | | | | ns | ns |
| N | | | | | ns |
| Nm | | | | | |

**Supplementary Table 3:** Multiple comparisons between conditions at each timepoint were carried out and the results at 30 and 40 minutes post-whole cell breakthrough.

| | FL oTau | NFRAG oTau | NFRAG mTau |
|---|---|---|---|
| 0 mins vs 20 mins | ** | * | ns |
| 0 mins vs 30 mins | ** | ** | * |
| 0 mins vs 40 mins | **** | **** | ** |

**Supplementary Table 4:** Action potential height was significantly reduced for FL oTau, NFRAG oTau and NFRAG mTau, but not vehicle or CFRAG oTau. This data is in full agreement with the conclusions in the manuscript.

**Action potential width**

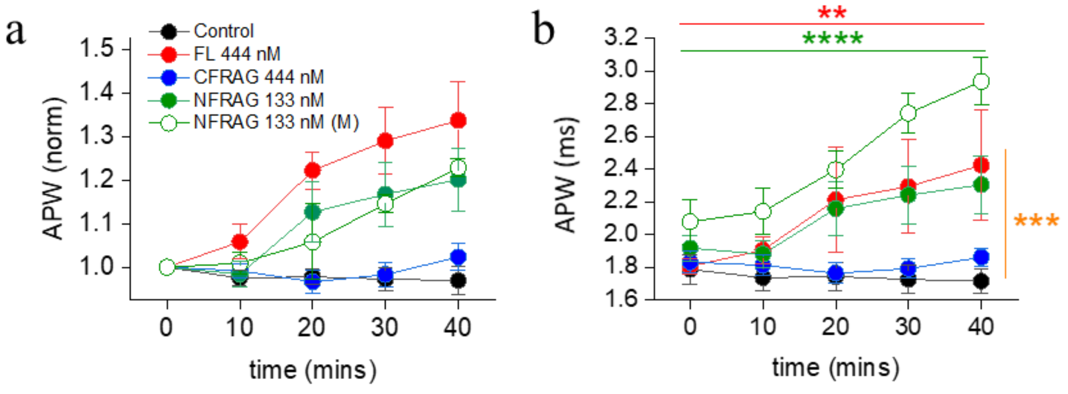


**Supplementary Figure 3: Changes to action potential width over time.** Data for all 5 conditions is plotted over time (mean ± SEM) both normalised (**a**), to allow changes over time to be clearly observed and non-normalised (**b**). 2-way ANOVA analysis was carried out on non-normalised data for all groups (V) Vehicle; (FL) Full length oTau; (C) CFRAG oTau; (N) NFRAG oTau; (Nm) NFRAG mTau. There was no statistical difference at time 0 between any of the conditions, in agreement with the manuscript. A significant difference was observed between the different conditions (F (4, 275) = 18.47, p =0.0002) and over time (F (4, 275) = 5.806, p < 0.0001). Error bars represent standard error of the mean (SEM).

| **30 mins** | V | FL | C | N | Nm |
|---|---|---|---|---|---|
| V | | ** | ns | * | **** |
| FL | | | * | * | * |
| C | | | | * | **** |
| N | | | | | * |
| Nm | | | | | |

| **40 mins** | V | FL | C | N | Nm |
|---|---|---|---|---|---|
| V | | *** | ns | ** | **** |
| FL | | | ** | * | * |
| C | | | | * | **** |
| N | | | | | ** |
| Nm | | | | | |

**Supplementary Table 5:** Multiple comparisons between conditions at each timepoint were carried out and the results at 30 and 40 minutes post-whole cell breakthrough.

| | FL oTau | NFRAG oTau | NFRAG mTau |
|---|---|---|---|
| 0 mins vs 30 mins | * | ns | ** |
| 0 mins vs 40 mins | ** | * | **** |

**Supplementary Table 6:** Action potential width was significantly increased for FL oTau, NFRAG oTau and NFRAG mTau, but not vehicle or CFRAG oTau. This data is in full agreement with the conclusions in the manuscript.

**Rheobase**

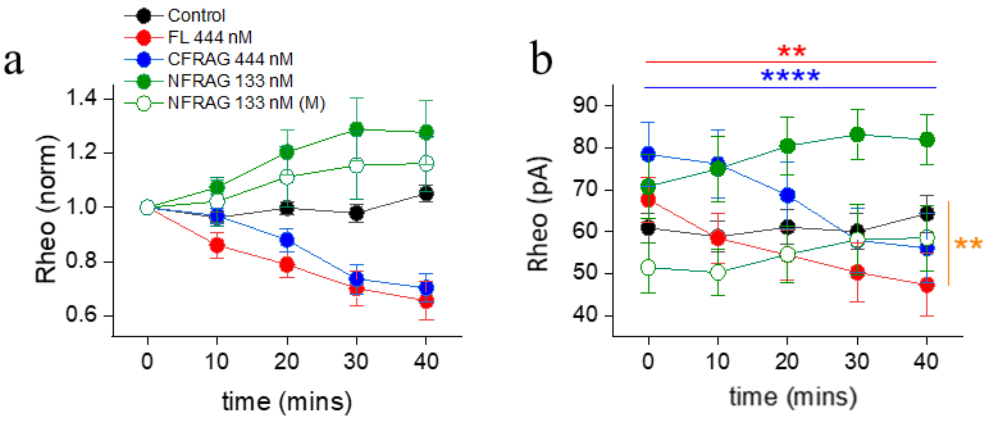


**Supplementary Figure 4: Changes to rheobase over time.** Data for all 5 conditions is plotted over time (mean ± SEM) both normalised (**a**), to allow changes over time to be clearly observed and non-normalised (**b**). 2-way ANOVA analysis was carried out on non-normalised data for all groups (V) Vehicle; (FL) Full length oTau; (C) CFRAG oTau; (N) NFRAG oTau; (Nm) NFRAG mTau. There was no statistical difference at time 0 between any of the conditions, in agreement with the manuscript. A significant difference was observed between the different conditions over time (F (4, 275) = 11, p < 0.0001). Error bars represent standard error of the mean (SEM).

| **30 mins** | V | FL | C | N | Nm |
|---|---|---|---|---|---|
| V | | ns | ns | * | ns |
| FL | | | | *** | ns |
| C | | | | ** | ns |
| N | | | | | ** |
| Nm | | | | | |

| **40 mins** | V | FL | C | N | Nm |
|---|---|---|---|---|---|
| V | | ns | ns | ns | ns |
| FL | | | ns | *** | ns |
| C | | | | ** | ns |
| N | | | | | * |
| Nm | | | | | |

**Supplementary Table 7:** Multiple comparisons between conditions at each timepoint were carried out and the results at 30 and 40 minutes post-whole cell breakthrough.

| | FL oTau | CFRAG oTau |
|---|---|---|
| 0 mins vs 30 mins | ns | * |
| 0 mins vs 40 mins | ** | * |

**Supplementary Table 8:** Rheobase was significantly reduced for FL-oTau and CFRAG oTau in agreement with the conclusions of the manuscript, but unaltered in vehicle, NFRAG oTau or NFRAG mTau.

**Spike threshold**

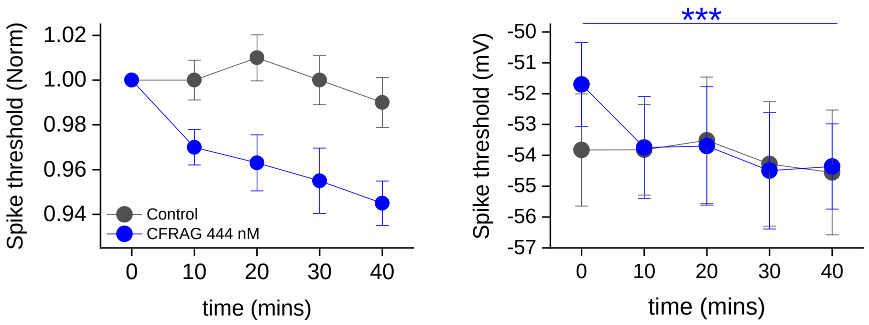


**Supplementary Figure 5: Tau-mediated change in spike threshold.** Control and CFRAG oTau 444 nM data are plotted over time (mean ± SEM) both normalised (**a**), to allow changes over time to be clearly observed and non-normalised (**b**). Analysis of spike threshold was carried out on the non-normalised data. There was no statistical difference at time 0 between the conditions, in agreement with the manuscript. Error bars represent standard error of the mean (SEM).

| | Control | $C^{FRAG}$ oTau |
|---|---|---|
| 0 mins vs 20 mins | ns | ** |
| 0 mins vs 30 mins | ns | ** |
| 0 mins vs 40 mins | ns | *** |

**Supplementary Table 9:** Spike threshold was significantly reduced CFRAG oTau over time (by 20 mins), reflecting an increase in excitability, which agrees with the conclusions of the manuscript.

**Spike onset**

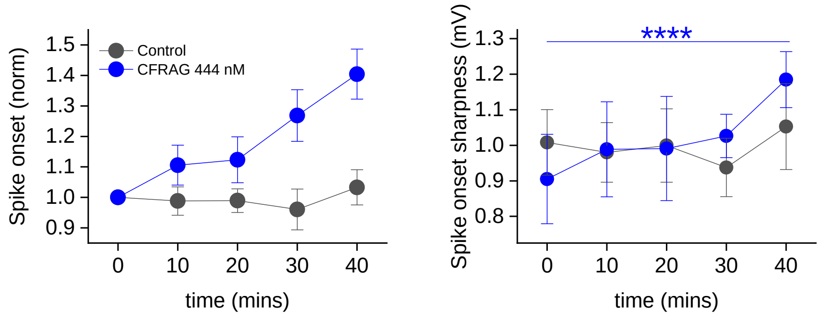


**Supplementary Figure 6: Tau mediated changes to spike onset.** Control and CFRAG oTau 444 nM data are plotted over time (mean ± SEM) both normalised (**a**), to allow changes over time to be clearly observed and non-normalised (**b**). Analysis of spike onset was carried out on the non-normalised data. There was no statistical difference at time 0 between the conditions, in agreement with the manuscript. Error bars represent standard error of the mean (SEM).

| | Control | $C^{FRAG}$ oTau |
|---|---|---|
| | | |
| 0 mins vs 30 mins | ns | ** |
| 0 mins vs 40 mins | ns | **** |

**Supplementary Table 10:** Spike threshold was significantly reduced CFRAG oTau over time (by 30 mins), reflecting an increase in excitability, which agrees with the conclusions of the manuscript. It was unaltered in control recordings.

**Sodium channel data**

Voltage gated sodium currents in acute slices were recorded using an adaptation of a published protocol, based on the use of a pre-pulse to inactivate axonal channels (Milescu et al., 2010). This protocol involved a number of modifications including altering both the internal and external solutions as well as performing the experiments at room temperature to slow the channel kinetics.

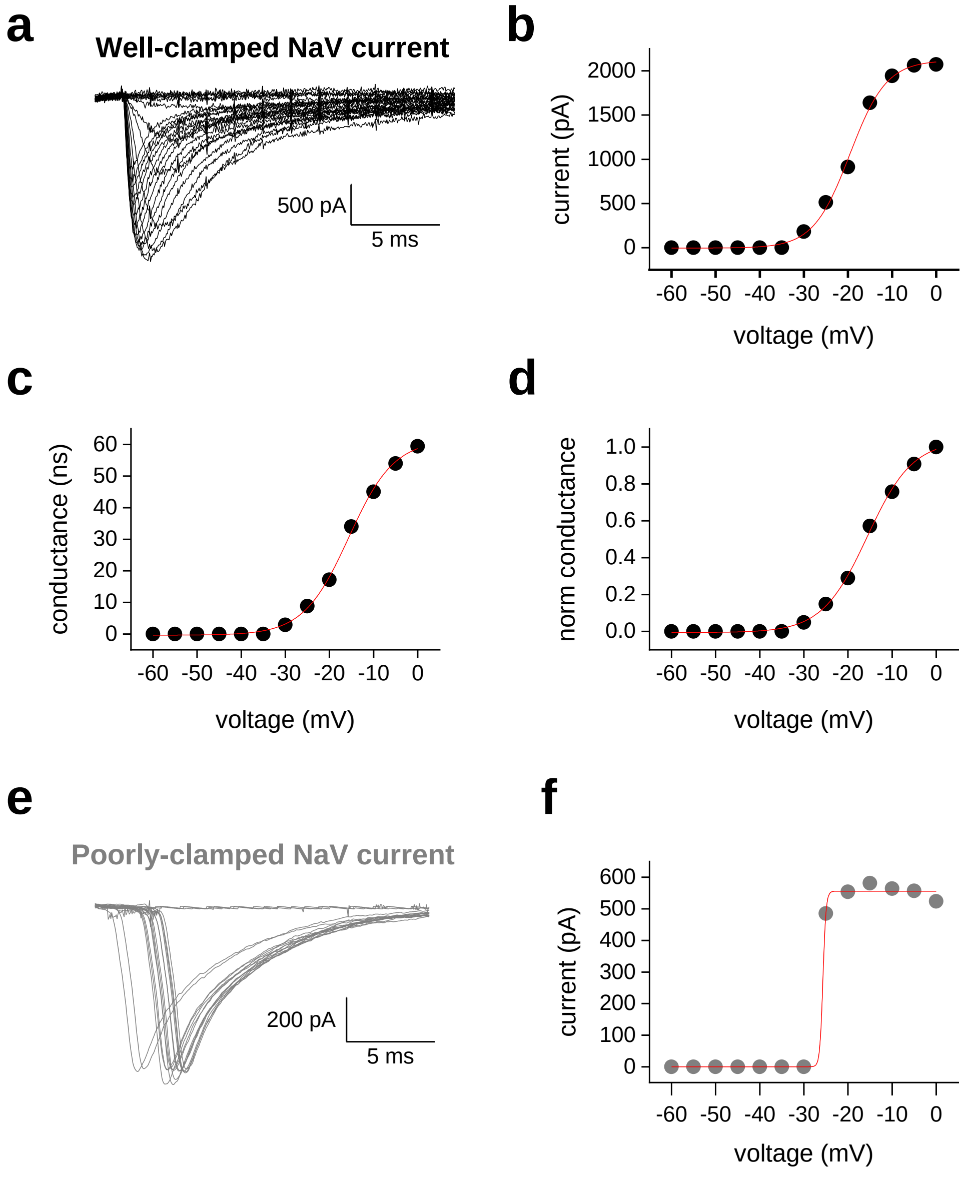


**Supplementary Figure 7:** As can be observed in (**a**) the currents recorded were well clamped under these conditions and could be reliably fit with a Boltzmann (**b**). There fit is unaffected by representing the data as conductance (**c**) or by normalising the data for easier comparison of conditions (**d**). For reference an early experiment performed at 30 degrees is shown (**e**) where voltage clamp has not been achieved, which is reflected in the poor fit to Boltzmann in (**f**).

**Bridge balance / series resistance measurements**

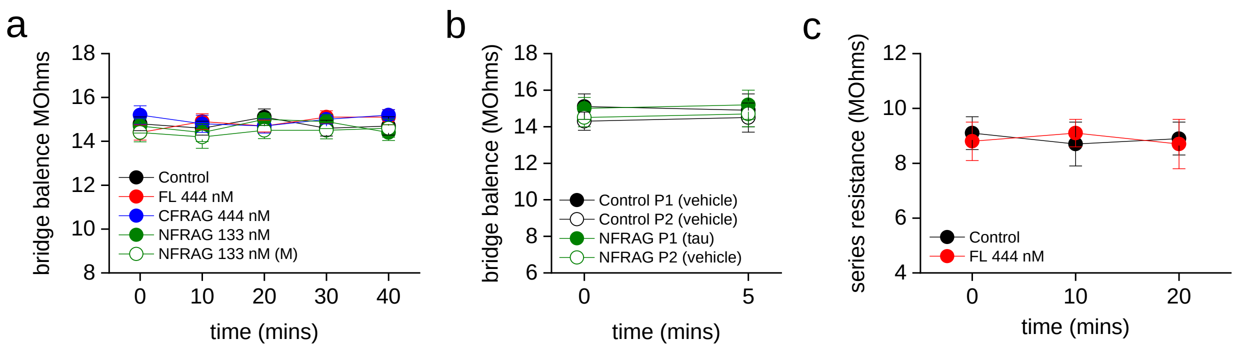


**Supplementary Figure 8: Bridge balance and series resistance measurements for all recordings over time.** A) Bridge balance measurements for all measured conditions over time, data is shown as mean and SEM. There was no change in bridge balance over the period of recording. B) Bridge balance measurements for the experiments presented in figure 5 for the double pipette analysis. In control recordings both pipettes had vehicle solution in. In the NFRAG recordings, one pipette had NFRAG tau while the other had vehicle solution. There was no change in bridge balance over the period of recording. C. Series resistance measurements for the experiments presented in figure 7 (sodium channel recordings). No change in series resistance was observed over the period of recording for either control or tau cells. Error bars represent standard error of the mean (SEM).
