# Supplementary material for: Truncating tau reveals different pathophysiological actions of oligomers in single neurons
Source: Commun Biol. 2021 Nov 4;4:1265. doi: 10.1038/s42003-021-02791-x (PMC8569149; doi:10.1038/s42003-021-02791-x)
Supplement: Supplementary file 3 — Description of Additional Supplementary Files [file 42003_2021_2791_MOESM3_ESM.pdf]

Description of Additional Supplementary Files

File name: Supplementary Data 1.
Description: Source data for all charts in manuscript.
